# Implementation of a Diversity Committee to Improve Structural Inclusion in an Emergency Medicine Residency

**DOI:** 10.30476/JAMP.2021.93492.1533.

**Published:** 2022-04

**Authors:** JESSICA BOD, DOWIN BOATRIGHT

**Affiliations:** 1 Department of Emergency Medicine, Yale School of Medicine, New Haven CT, USA

**Keywords:** Diversity, Recruitment, Emergency medicine

## Abstract

**Introduction:**

To describe structural changes that can be made in an emergency medicine residency program to increase diversity and foster an inclusive environment.

**Methods:**

We established a diversity committee to introduce several simultaneous structural changes focusing on resident recruitment, education and engagement. Some of these changes include establishment of a scholarship to recruit visiting students from diverse backgrounds and a second look weekend for minority applicants. Others focused on ensuring residency didactics, were inclusive and addressed topics pertaining to diversity in emergency medicine.

**Results:**

We increased the number of minority residents underrepresented in medicine matching in our program from zero to between 2 and 4 annually. We increased the percentage of women matching in our program from 33% to 47%. We worked with residency leadership to increase the number of didactics focusing on diversity and inclusion.

**Conclusions:**

Implementation of a Diversity Committee in emergency medicine training programs can be an important tool to improve diversity on a structural level.

## Introduction

The benefits of a diverse physician workforce are well known. There is evidence that patients from racial and ethnic minority backgrounds have better interactions with physicians from similar backgrounds with respect to patient-centered outcomes, and that physicians who are underrepresented in medicine (URiM) are more likely to meet the needs of minority and underserved communities ( 1 , 2 ). Prioritizing diversity and inclusion in the recruitment of physicians serves an important role in correcting the harm done by decades of exclusionary policies that prevented women and minorities from becoming physicians ( 2 ). In the 2014 and 2015 academic years, our emergency medicine (EM) program did not match any residents identified as URiM. In those same years, only one third of the residents matched in our program were women. We therefore sought innovative solutions that would improve the diversity of our resident cohort. 

In 2019, the Accreditation Council for Graduate Medical Education (ACGME) established Common Program Requirements focusing on improving diversity and inclusion in residency training programs ( 3 ). These include “practices that focus on mission-driven, ongoing, systematic recruitment and retention of a diverse and inclusive workforce of residents” ( 4 ). In spite of this guidance, in the specialty of emergency medicine only 36% of residents are women, and only 13% are from racial or ethnic groups that are underrepresented in medicine (i.e. Black, Hispanic/Latino, American Indian, Pacific Islander, Alaska Native or Native Hawaiian). This demographic makeup of EM residents has not changed over the last 5 years ( 5 ). Data on sexual and gender minorities were not collected. 

There are many structural barriers that must be addressed to create, support, and sustain a diverse and inclusive residency program. In 2009, the Council of Emergency Medicine Residency Directors (CORD) recommended 8 key recruitment strategies in order to increase diversity in EM. These strategies include active recruitment of students from diverse backgrounds, validation of the importance of applicants meeting with residents and faculty from underrepresented groups during recruitment, development of curricula to address topics on diversity equity and inclusion, and celebration of successful institutional diversity initiatives ( 6 , 7 ). In 2019, CORD made additional recommendations including dedication of funds to the recruitment and retention of URiM residents, participation in pipeline activities to increase the proportion of URiM trainees, and addressing the climate of inclusion within EM residency programs ( 6 ). Some EM residency programs have described successful implementation of initiatives to recruit residents who are URiM, focusing on pipeline programs, interview day initiatives and a holistic evaluation of residency applicants ( 8 , 9 ). 

Individuals identifying as racial/ethnic minorities, women, and sexual/gender minorities face increased discrimination during medical training, including overt mistreatment and implicit bias ( 10 , 11 ). It is important for these trainees to have institutional structures in place to share experiences, foster social connections, and to promote an inclusive environment ( 12 ). When only one third of our matched residents were women and none of our matched residents identified as URiM in the 2014 and 2015 academic years, we recognized that we needed to address diversity by making meaningful and sustainable structural changes in our program. In 2016, we instituted a Diversity Committee tasked with recruiting a more diverse cohort of residents as well as establishing structures within the program to provide an inclusive educational environment.

## Methods

 The Diversity Committee was established to introduce structural changes that would increase diversity and inclusion in our EM residency program. Led by two faculty co-chairs, one resident chair and open to all residents who wanted to participate, the committee discussed, developed, and instituted several simultaneous initiatives intended to synergistically recruit and support a more diverse cohort of residents. 

Based on the success of other EM diversity recruitment programs, and the personal experiences of our resident members, the Diversity Committee established a multifaceted effort to recruit a diverse class of residents at different stages of the recruitment process ( 8 , 9 ). We recognized that applicants to EM residency programs consider previous rotations with a department to be an important factor in deciding rank list order ( 13 ) and that URiM applicants value programs’ visible commitment to diversity ( 14 ). Our Diversity Committee resident members gave their input as to what type of outreach was most impactful for them personally when they were applying for residency. We then established a recruitment stragegy that would allow URiM and women applicants multiple points of contact with our department. We reviewed the recruitment materials provided to applicants to ensure that commitment to diversity was explicitly highlighted during interview days. The Diversity Committee also began to think critically about how diverse residents were represented, included, and supported within the educational frameworks of the residency. We solicited input from the resident members of the committee to identify areas in which our Diversity Committee could work with residency leadership to make meaningful changes in our didactic curriculum, simulation program, and other educational structures. 

We successfully petitioned the emergency department chair to provide a budget to defray the cost of travel and lodging associated with a visiting sub-internship. Five scholarships of $1500 were awarded annually to URiM visiting students. Applicants for the scholarship were asked to provide a CV and a written statement explaining their interest in leadership and academics. Scholarship applications were reviewed by a committee consisting of the Diversity Committee faculty and resident chairs, and the residency program director. Scholarships were then awarded on a rolling basis. 

Residents on our Diversity Committee identified sustained and frequent outreach emphasizing a commitment to diversity equity and inclusion to be an important factor in their own decisions about where to apply for residency training and where they wanted to match. We therefore decided to begin our outreach early- well before residency applications were completed. We selected residents on the Diversity Committee to represent our program at the Student National Medical Association and to speak at events sponsored by EM interest groups at medical schools affiliated with historically Black colleges and universities such as Howard University College of Medicine and Meharry Medical College. Our goal at this stage of recruitment was to attract visiting students and residency applicants identifying as URiM and to clearly establish our program’s commitment to recruiting a diverse cohort of residents. 

Any student doing a sub-internship at our program, whether as a URiM scholarship recipient or not, was informed via email of our program’s commitment to diversity, equity and inclusion and was invited to seek information pertaining to life as a racial, ethnic, gender and/or sexual minority in our program and city. Resident volunteers met with rotators who self-identified as women, URiM and sexual/gender minorities to provide information about the inclusive culture fostered by our program. We emphasized that these meetings were informal and unrelated to assessment or grading during the sub-internship. 

After being selected to interview, all applicants to our EM residency program were also invited via email to seek information pertaining to life as a racial, ethnic, gender and/or sexual minority from the residents. Interested applicants were matched with resident volunteers who could share personal experience about their individual concerns either via email, on the phone, or during the interview day. These meetings were also informal, and sometimes would take place at the casual dinner prior to the interview day. Again, we emphasized that these meetings were for the purpose of providing information and fostering connections and that they were not part of the interview assessment. 

Prior to the establishment of the Diversity Committee, the materials and presentations provided on the interview day did not explicitly emphasize a commitment to diversity and inclusion in our program, and focused mainly on the details of the training program and on academic research. Our Diversity Committee quickly identified this as a missed opportunity for program leadership to build commitment to diversity into the structure of the interview day and to provide a clear message to diverse applicants that their identities were valued on an institutional level. We revamped the materials provided to interviewees to include information about the minority house staff office, LGBTQ life in our city, and the importance and contributions of women leaders in our department. 

Our resident Diversity Committee members identified the interval between interviews and the finalization of residency match lists as an important time to re-emphasize an institutional commitment to diversity. Additional departmental funds were secured to invite interested URiM students for a second look after interviewing but before the match rank order lists were finalized. Programming for the second look included meeting with key departmental leadership such as the department chair, the residency program director, the assistant program directors and senior faculty members identifying as URiM. Applicants attending second look participated in a medical simulation activity at our Center for Medical Simulation, and were invited to shadow faculty working clinically in the emergency department. We also arranged for attendees to have dinner with the Diversity Committee resident and faculty members, and to attend a party hosted by the minority house staff association. 

Once recruited, it is important to ensure an inclusive learning environment in which a diverse group of residents can thrive. To that end, the Diversity Committee began to address structures within the educational milieu of the classroom and hospital. We worked closely with the associate program director in charge of residency didactics to identify areas related to diversity, equity and inclusion that were lacking in our curriculum, and expanded the didactic curriculum to include these areas structurally as a recurring curricular objective. We successfully incorporated implicit bias training into our curricula to mitigate the effect of implicit bias in our interactions as colleagues and clinicians. We also created a bystander intervention workshop so that allies could support colleagues and junior trainees when subjected to mistreatment related to race, ethnicity, gender, and sexual orientation. Speakers were invited to share their experiences as members of diverse groups. For example, we hosted a panel on the experience of transgender healthcare workers. Finally, we initiated a grand rounds lecture series focusing on diversity equity and inclusion. Subjects ranged from political advocacy as a physician, to trauma informed care in the emergency department, to the care of incarcerated populations. 

In addition to creating new didactic content focusing on diversity equity and inclusion, we also sought ways to make existing didactic offerings more inclusive. For example, we recognized the importance of including patients of diverse skin tones in dermatology lectures and ensured that the example slides of skin findings were inclusive. We incorporated subjects pertaining to diversity, equity and inclusion in healthcare into our monthly journal club, and are currently examining our simulation curriculum to ensure that same sex couples are represented in simulated cases requiring collateral information from a spouse. 

The Diversity Committee continually reassess the needs of the residency program based on input from committee members annually. New resident chairs and junior members bring in new ideas for improving diversity recruitment and inclusivity in our residency, while the faculty co-chairs provide ongoing experience, institutional memory, and guidance when liaising with departmental and residency leadership. 

## Results

Our Diversity Committee was created, in part, due to the low numbers of URiM and women residents in our 2014 and 2015 classes. Between 2016 and 2019 we awarded 19 scholarships to support visiting rotators from underrepresented backgrounds, increasing our number of URiM visiting students from 2 to an average of 7 annually. We have recruited 5 scholarship recipients to our program in total, and have increased our number of URiM residents from zero in 2014 and 2015 to between 2 and 4 annually for the last four years. As of November 2020, URiM residents comprise 18% of our residency cohort. In 2014 and 2015, 33% of matched residents were women. Since establishing the Diversity Committee, 47% of matched residents are women. Data about sexual and gender minorities has not been collected. 

Prior to 2016 we did not have any didactic conferences in our residency focusing explicitly on diversity and inclusion. Since the establishment of the Diversity Committee, we have had four grand rounds speakers, one panel discussion and one journal club focusing on diversity and inclusion. We have also provided implicit bias training and bystander intervention training for the entire residency and faculty. The Diversity Committee continually scrutinizes the didactic program to ensure that educational programming is structurally inclusive, even when the content does not explicitly pertain to diversity equity and inclusion. 

## Discussion

Many residency programs struggle to recruit diverse residency classes. Developing diversity where there is little or no diversity is a challenging task. Programs with existing resident and faculty diversity may be more attractive to underrepresented minority and female residency applicants. This leaves programs that have not already established diversity at a disadvantage in recruiting diverse applicants, and lack of diversity becomes self-perpetuating. Our Diversity Committee initiatives were successful in mitigating the effects of two application cycles in which no URiM residents were recruited and only 33% of residents recruited were women. These efforts have proven to be sustainable over four years. 

Program leaders seeking to improve their resident diversity will need buy-in from key stakeholders. The active participation of women, URiM and sexual/gender minority residents on our Diversity Committee was important in identifying what outreach strategies to implement, and what information about our program to emphasize to recruit diverse residency applicants. They also gave key input as to what changes should be made to our didactic curriculum to foster a more inclusive residency program. Departmental leaders are also important stakeholders. In order to secure funding to implement our recruitment strategies, we made a formal presentation to our department chair. We presented published evidence showing the success of other recruitment programs and continue to provide annual data documenting our success to demonstrate the need for ongoing funding. We also worked closely with the associate program director in charge of resident conference to make changes to the didactic curriculum. We emphasized shared common goals of fostering diversity equity and inclusion in the residency to convey the importance of dedicating many hours of conference time to these curricular initiatives. 

Programs without existing diversity can look to university or hospital-based groups, such as the minority house staff association, to partner with in their recruitment efforts. Our partnership with the minority house staff association at our hospital allowed second look participants to appreciate a wider institutional commitment to supporting URiM residents outside of our individual EM department. 

Next steps for our Diversity Committee include a focus on creating and supporting opportunities for advocacy and community outreach, an area identified as important by our residents. The COVID-19 pandemic has impacted some of our recruitment strategies. We are currently looking at what virtual outreach strategies have been most effective in the 2021 academic year and how they might be carried forward to support diversity recruitment at multiple stages of the application process. We are also discussing ways to sensitively recruit sexual and gender minorities, a group that has not typically been the focus of pipeline programs. We anticipate that it may be more difficult to collect data on the success of recruitment initiatives for sexual and gender minorities, since these demographics are not routinely collected on residency applications. 

## Conclusion

Institution of a Diversity Committee to make structural changes in residency programs can increase recruitment of URiM and women residents. Our Diversity Committee has demonstrated sustained success in recruiting a more diverse cohort of residents over four years. The approach of our Diversity Committee can be generalized to residencies in other specialties to increase recruitment and inclusion of a diverse cohort of residents. More work is needed to address the way recruitment is changing in response to COVID-19 and to sensitively identify and recruit sexual and gender minorities. 

## Conflict of Interest:

None Declared.
